# Exploring candidate biomarkers for rheumatoid arthritis through cardiovascular and cardiometabolic serum proteome profiling

**DOI:** 10.3389/fimmu.2024.1333995

**Published:** 2024-02-14

**Authors:** Laura Cuesta-López, Alejandro Escudero-Contreras, Yas Hanaee, Carlos Pérez-Sánchez, Miriam Ruiz-Ponce, Julio Manuel Martínez-Moreno, Eva Pérez-Pampin, Antonio González, Chamaida Plasencia-Rodriguez, Ana Martínez-Feito, Alejandro Balsa, Clementina López-Medina, Lourdes Ladehesa-Pineda, Marta Rojas-Giménez, Rafaela Ortega-Castro, Jerusalem Calvo-Gutiérrez, Chary López-Pedrera, Eduardo Collantes-Estévez, Iván Arias-de la Rosa, Nuria Barbarroja

**Affiliations:** ^1^ Rheumatology Service, Department of Medical and Surgical Sciences, Maimonides Institute for Research in Biomedicine of Cordoba (IMIBIC), Reina Sofia University Hospital, University of Cordoba, Córdoba, Spain; ^2^ Scientific department, Cobiomic Bioscience S.L, Cordoba, Spain; ^3^ Department of Cell Biology, Immunology and Physiology, Agrifood Campus of International Excellence, University of Córdoba, Córdoba, Spain; ^4^ Experimental and Observational Rheumatology and Rheumatology Unit, Instituto de Investigación Sanitaria - Hospital Clínico Universitario de Santiago (IDIS), Santiago de Compostela, Galicia, Spain; ^5^ Rheumatology Department, Instituto de Investigación Hospital Universitario La Paz (IdiPAZ) Institute for Health Research, La Paz University Hospital, Madrid, Spain

**Keywords:** rheumatoid arthritis, methotrexate, tofacitinib, biomarkers, proximity extension assay (PEA), Olink

## Abstract

**Introduction:**

RA patients are at higher risk of cardiovascular disease, influenced by therapies. Studying their cardiovascular and cardiometabolic proteome can unveil biomarkers and insights into related biological pathways.

**Methods:**

This study included two cohorts of RA patients: newly diagnosed individuals (n=25) and those with established RA (disease duration >25 years, n=25). Both cohorts were age and sex-matched with a control group (n=25). Additionally, a longitudinal investigation was conducted on a cohort of 25 RA patients treated with methotrexate and another cohort of 25 RA patients treated with tofacitinib for 6 months. Clinical and analytical variables were recorded, and serum profiling of 184 proteins was performed using the Olink technology platform.

**Results:**

RA patients exhibited elevated levels of 75 proteins that might be associated with cardiovascular disease. In addition, 24 proteins were increased in RA patients with established disease. Twenty proteins were commonly altered in both cohorts of RA patients. Among these, elevated levels of CTSL1, SORT1, SAA4, TNFRSF10A, ST6GAL1 and CCL18 discriminated RA patients and HDs with high specificity and sensitivity. Methotrexate treatment significantly reduced the levels of 13 proteins, while tofacitinib therapy modulated the expression of 10 proteins. These reductions were associated with a decrease in DAS28. Baseline levels of SAA4 and high levels of BNP were associated to the non-response to methotrexate. Changes in IL6 levels were specifically linked to the response to methotrexate. Regarding tofacitinib, differences in baseline levels of LOX1 and CNDP1 were noted between non-responder and responder RA patients. In addition, response to tofacitinib correlated with changes in SAA4 and TIMD4 levels.

**Conclusion:**

In summary, this study pinpoints molecular changes linked to cardiovascular disease in RA and proposes candidate protein biomarkers for distinguishing RA patients from healthy individuals. It also highlights how methotrexate and tofacitinib impact these proteins, with distinct alterations corresponding to each drug’s response, identifying potential candidates, as SAA4, for the response to these therapies.

## 1 Introduction

Cardiovascular disease (CVD) has been identified as the primary contributor to premature mortality and sudden death among patients with rheumatoid arthritis (RA). This population has an incidence of CVD at least two times greater than the general population, with cardiovascular mortality being a major cause of death, accounting for 40-50% of RA-related deaths (1). This may be partly attributed to the high prevalence of traditional CVD risk factors (e.g., hypertension, hypercholesterolemia, type II diabetes mellitus, obesity) in conjunction with chronic systemic inflammation (1, 2). In this sense, the prevalence of metabolic syndrome in RA patients is significantly higher (around 30%) compared to the general population. Metabolic Syndrome has been linked to a three-fold increase in the risk of atherosclerotic cardiovascular disease (CVD) (3, 4). Our recent research investigated whether RA-associated inflammatory activity could explain the observed defects in glucose and lipid metabolism in these patients. Our results illustrate that alterations in glucose and lipid homeostasis associated with RA depend on the degree of inflammation, with adipose tissue inflammation identified as the initial target leading to insulin resistance and molecular alterations. Therefore, therapeutic strategies targeting tighter control of inflammation and flare-ups could normalize and/or prevent RA-associated metabolic alterations (5). Circulating proteins have been used as biomarkers of various pathologies for many years. Currently, different technologies are used to measure and analyze proteins in serum or plasma, but accurately measuring and interpreting the complete protein content in a large number of samples is a major challenge. To address this, the Olink Proximity Extension Assay (PEA) has been developed—an advanced, high-throughput method that analyzes up to 92 protein biomarkers with exceptional sensitivity and precision using oligonucleotide-labeled antibodies (6, 7). Despite this, there are still few studies that analyze the serum proteome in RA patients. Early diagnosis of RA is essential for the optimal treatment. According to EULAR recommendations, synthetic DMARDs such as methotrexate should be used as first-line treatment. When the first treatment fails, EULAR recommends that patients with risk factors for severe disease and a high inflammatory burden should receive biologic DMARDs. The introduction of biological therapies has significantly improved the management of RA, allowing for the reduction of symptoms, the prevention of rapid radiological deterioration, and an improvement in the quality of life. On the other hand, Tofacitinib is a targeted synthetic DMARD, reversible, competitive inhibitor that works by blocking the adenosine triphosphate (ATP) binding site in the catalytic cleft of JAK1, JAK2, JAK3 and TYK2 and it has been approved for the treatment of RA in many countries. It has been shown to reduce HAQDI and promote ACR20 responses in patients that had an inadequate response to conventional synthetic DMARDs or TNFα inhibitors. However, its efficacy in improving CV risk and reducing cardiovascular risk factors, including mediators of metabolic syndrome, is yet to be determined. This study aims to evaluate the changes in the cardiometabolic and cardiovascular serum proteome in two cohorts of active RA patients: newly diagnosed (naïve- treated) and those with well-established disease and to analyze the association with the clinical characteristics. Furthermore, the study analyses the modulation of the levels of these proteins by methotrexate and tofacitinib.

## 2 Materials and methods

### 2.1 Patients

A cross-sectional study was conducted on 50 patients with RA the from the Rheumatology Department of the Hospital Universitario Reina Sofia in Cordoba, the Hospital Universitario La Paz in Madrid and the Hospital Clínico Universitario de Santiago de Compostela in Santiago de Compostela, Spain. The patients fulfilled the American College Rheumatology 2010 criteria for RA and were divided in 2 independent cohorts: a cohort of 25 newly diagnosed RA patients (disease duration = 0 years) and a second cohort of 25 patients with established RA (disease duration > 20 years). None of the patients had a history of previous CV events (ischemic heart disease, stroke, peripheral arterial disease or heart failure). Additionally, 25 age- and -sex matched healthy donors (HDs) were included as a control group, none of which had a history of other autoimmune diseases or cardiovascular diseases/events. All recruited subjects provided written informed consent, which was specifically approved by the hospital ethics committee (ethics committee of the Reina Sofia Hospital, the University Clinical Hospital of Santiago de Compostela, and La Paz University Hospital). Demographic and clinical data were collected, including disease duration and DAS-28 (disease activity score 28), inflammation (CRP, mg/L), and levels of autoantibodies (rheumatoid factor and ACPAs). To assess disease activity, we computed the modified Disease Activity Score including a 28-joint count (DAS28), incorporating information on tender and swollen joints, the patient’s global assessment of disease activity on a visual analogue scale (VAS), and acute phase response. As we utilized CRP values (8), it will be subsequently referred to as DAS28-CRP. On the other hand, a longitudinal study was carried out in the 25 newly-diagnosed RA patients which were treated with methotrexate and the 25 RA patients with established disease that were treated with tofacitinib (5mg twice daily) in combination with conventional DMARDs, both according to the daily clinical practice for 6 months. Treatment response was determined after 6 months of treatment by the change in DAS28 (CRP) based on European Alliance of Associations for Rheumatology (EULAR) criteria (9). It considers that a patient has a good response to treatment when, having a DAS28 ≤3.2, it is reduced by at least 1.2. Moderate responders include three scenarios: patients who have a DAS28 ≤3.2 and the decrease in activity is between 0.6 and 1.2; those having a DAS28 >3.2 ≤ 5.1, the diminution is higher than 0.6; and those with a DAS28 >5.1 and a reduction higher than 1.2. The occurrence of cardiovascular events was recorded. At baseline and follow-up visits, peripheral blood samples were collected.

#### 2.1.1 Measurements

##### 2.1.1.1 Blood sample collection and isolation of serum

Peripheral venous blood samples were collected from RA patients before and after treatment and from HDs. Samples were collected early in the morning and after an 8-hour fasting period. The samples were centrifuged for 10 minutes at 3500 rpm and at room temperature to obtain the serum, which was then aliquoted and stored at -80°C until use.

##### 2.1.1.2 Proximity extension assay

Serum samples from the baseline and follow-up visits were subjected to high-throughput analysis of 184 proteins, 92 of which were cardiometabolic-associated and 92 cardiovascular disease-related. This analysis was conducted using proximity extension immunoassay (PEA) provided by Olink Proteomics, Uppsala, Sweden, and was performed in a 96-well plate format by Cobiomic Bioscience S.L, Cordoba, Spain. The PEA is a dual-recognition immunoassay, that uses matched antibodies each of them labeled with unique DNA oligonucleotides, to simultaneously bind to a target protein in solution. This allows the two antibodies to converge and their DNA oligonucleotides to hybridize, serving as a template for a DNA polymerase-dependent extension step. This creates a double-stranded DNA “barcode” which is unique for the specific protein. The hybridization and extension are followed by PCR amplification. The resulting concentration of the PCR product is directly proportional to the initial concentration of the target protein. The relative levels of proteins were reported on an arbitrary Log2-based NPX (normalized protein expression) scale. The samples were completely randomized and distributed across two plates, maintaining the representation of groups/treatments in proportion to the study. Within each 96-plex panel, there are 96 assays, including four internal controls for quality control, systematically monitoring various stages of the process in every sample. Additionally, interplate controls are incorporated to compute normalized expression (NPX) values, along with negative controls and a duplicated external control. In the intensity normalization process, data were adjusted so that the median NPX for a protein on each plate aligned with the overall median. This ensured that each plate was adjusted to have the same median for all assays across the two plates, thereby enhancing the reliability and consistency of the analysis.

##### 2.1.1.3 Statistical analysis

All data analyses were performed using SPSS statistical software package (Iberica, Madrid, Spain), GraphPad Prism9 (version 9.0.1) and MetaboAnalyst 5.0. Graphical representation of the statistical analysis is carried out using Prism9 (version 9.0.1) and MetaboAnalyst 5.0 software. Data in the text, figures and tables were expressed as the mean ± standard deviation (SD). Normality of variables was assessed using the Kolmogorov-Smirnov test. Clinical characteristics were compared using Student’s unpaired t-test for parametric data and the Mann-Whitney sum test for non-parametric data. Paired samples within the same subjects were compared using the paired Student’s t test. Correlations were assessed using Pearson correlation between variables. Chi-square tests were performed to analyze qualitative data. A volcano plot, featuring Benjamini-Hochberg adjusted false discovery rates, provided a nuanced visualization of the differential expression of 184 proteins across distinct study groups. Complementing this, Venn diagram was employed to discern the intersection of commonly altered proteins among the groups, shedding light on shared molecular signatures. To delve deeper into the differences among HDs, early RA, and established RA, an analysis of variance (ANOVA) was executed, with subsequent Bonferroni adjustments ensuring robustness in multiple comparisons in the commonly altered proteins. Receiver operating characteristic (ROC) curves, representing the true positive rate (sensitivity) versus false positive rate (1-specificity) at various thresholds, and area under the curve (AUC) analysis were used to determine sensitivity, specificity and corresponding cut-off values. Statistical significance levels were designated as follows: (****) <0.0001 *p*-value; (***) <0.001 *p*-value; (**) <0.01 *p*-value; (*) <0.05 *p*-value. This convention was applied in instances where specific *p*-values were not explicitly provided in the graphs.

## 3 Results

### 3.1 RA patients show an altered cardiometabolic and CVD profile: candidate biomarkers in human serum

RA patients with less than two years of disease duration (newly diagnosed RA) showed similar age, sex and disease activity compared to RA patients with stablished disease. In addition, RA patients with stablished disease showed a mean disease duration of 37.48 ± 11.79 years and significantly elevated levels of C-reactive protein compared to patients with newly diagnosed RA (**Table 1**). CVD risk factors were elevated in RA compared HDs.

**Table 1:** Descriptive clinical data of Rheumatoid Arthritis patients and healthy donors.

	HDs	Early RA	Established RA
**Size population**	25	25	25
**Female/male (%)**	78/22	75/25	70/30
**Age (years)**	58.55 ± 13.12	61.24 ± 12.65	63.75 ± 10.19
**Disease duration (years)**	–	0	37.48 ± 11.79^b^
Disease activity			
**DAS-28 (CRP)**	–	5.04 ± 1.20	4.82 ± 0.71
**CRP (mg/L)**	1.71 ± 1.94	1.48 ± 1.90	8.12 ± 7.39^a,b^
Autoimmunity profile			
**RF + (n)**	–	16	12
**ACPAs + (n)**	–	18	21
CVD risk factors			
**Arterial Hypertension (%)**	12	20	36^a^
**Obesity (%)**	12	16	36^a^
**Smoking habit (%)**	12	24^a^	24^a^
**Type 2 Diabetes Mellitus (%)**	0	8	12^a^
Metabolic profile			
**Glucose (mg/dL)**	85.06 ± 9.66	89.62 ± 11.89	94.30 ± 22.98
**Total-cholesterol (mg/dL)**	198.50 ± 24.19	190.25 ± 32.91	202.90 ± 9.66
**HDL-cholesterol (mg/dL)**	58.65 ± 19.06	55.78 ± 21.83	65.33 ± 12.27
**LDL-cholesterol (mg/dL)**	119.20 ± 23.44	115.38 ± 32.08	112.86 ± 32.22
**Tryglicerides (mg/dL)**	99.94 ± 50.54	140.41 ± 94.15	103.73 ± 41.08

Data are represented by mean ± SD. HDs, healthy donors; RA, rheumatoid arthritis; DAS, disease activity score; CRP, c-reactive protein; RF, rheumatoid factor; ACPAs, antibodies to citrullinated protein antigens. ^a^Significant differences respect to HDs, p value < 0.05; ^b^Significant differences respect to early RA, p value < 0.05.

**Figure 1:**
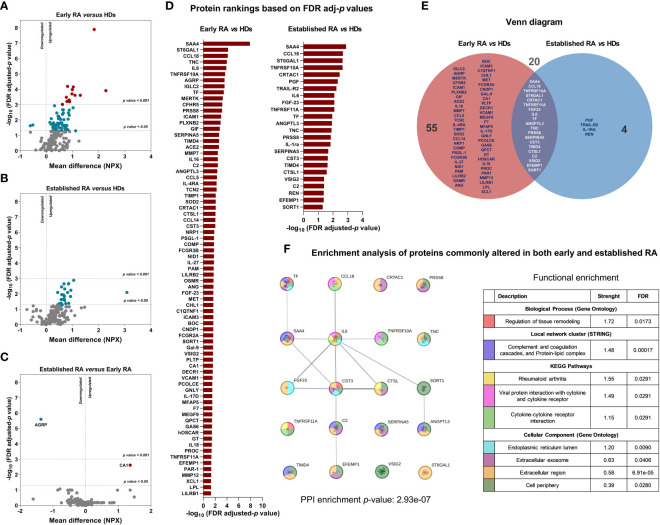
CVD and cardiometabolic serum proteome profile of RA patients in early and established disease. **(A)** Volcano plot of CVD and cardiometabolic proteome profile (184 proteins) in early RA (n=25) respect to HDs (n=25). **(B)** Volcano plot of CVD and cardiometabolic proteome profile (184 proteins) in established RA (n=25) respect to HDs (n=25). **(C)** Volcano plot of CVD and cardiometabolic proteome profile (184 proteins) in established RA (n=25) respect to early RA (n=25). **(D)** Significant protein rankings based on FDR adjusted *p*-values in early RA and established RA compared to HDs. **(E)** Venn diagram illustrating the proteins commonly altered in both early RA and established RA in comparison to HDs. **(F)** Enrichment analysis of proteins commonly altered in both early and established RA using STRING platform (version 12.0, STRING CONSORTIUM 2023). CVD, cardiovascular disease; RA, rheumatoid arthritis, HDs, healthy donors. Annotated names of abbreviated proteins are displayed in **Supplementary Table 1**.

To analyze the molecular profile that might be associated with CVD in RA patients, we evaluated the levels of 184 proteins related to cardiometabolism and CVD in the serum. Seventy-five proteins were significantly altered in early RA patients compared to HDs (**Figure 1A**) and 24 proteins were significantly increased in RA patients with established disease *vs* HDs (**Figure 1B**). In contrast, the comparison of the proteome profile between early and established disease revealed significant alterations in only 2 proteins (**Figure 1C**). The protein rankings based on FDR-adjusted *p*-values are depicted in **Figure 1D**. Additionally, a Venn diagram was utilized to identify commonly altered proteins in both early RA and established RA compared to HDs, revealing 20 proteins that were consistently affected in both conditions (**Figure 1E**). The altered levels of these proteins did not correlate with autoantibodies titers or positivity. We conducted an enrichment analysis to elucidate whether the identified proteins contribute to specific pathways beyond their recognized cardiovascular implications using STRING platform, particularly in the shared alteration pattern observed in both early and established RA disease (**Figure 1F**). The enrichment analysis revealed a significant protein-protein interaction enrichment (*p*-value: 2.93e-07), underscoring the biological relevance of the identified protein alterations. These proteins were found to play pivotal roles in diverse processes, including the regulation of tissue remodeling, complement and coagulation cascades, and the formation of protein-lipid complexes. Moreover, their implication in pathways directly associated with RA pathology was evident. Notably, these proteins exhibited interactions in viral protein networks and demonstrated associations with various cellular components, such as the endoplasmic reticulum lumen, extracellular exosome, extracellular region, and cell periphery.

**Figure 2:**
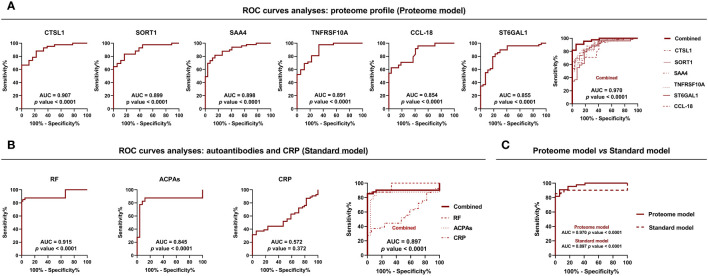
Prospective serum biomarkers with diagnostic potential in RA. **(A)** ROC curve analysis of proteins significantly altered in both early and established disease in the whole cohort of RA patients with the best capacity to identify RA (n=50) *vs* HDs (n=25) (Proteome model). **(B)** ROC curve analysis of commonly used diagnostic markers for RA, such as ACPAs, RF, and CRP (n=50) *vs* HDs (n=25) (Standard model). **(C)** Comparison of the ability to differentiate between RA patients and HDs using proteomic and standard models. RA, rheumatoid arthritis; HDs, healthy donors; ACPAs, antibodies to citrullinated protein antigens; RF, rheumatoid factor; CRP, C-reactive protein; ROC, receiver operating characteristic; AUC, area under the curve. Annotated names of abbreviated proteins (**Supplementary Table 1**).

Notable proteins in the shared alteration (early and established RA conditions) included SAA4, CCL18, TNFRSF10A, ST6GAL1, CRTAC1, TNFRSF11A, FGF23, IL6, TF, ANGPTL3, TNC, PRSS8, SERPINA5, CST3, TIMD4, CTSL1, C2, VSIG2, EFEMP1 and SORT1 (**Supplementary Figure 1**). Interestingly, elevated levels of CTSL-1 (AUC=0.907), SORT1 (AUC=0.899), SAA-4 (AUC = 0.898), TNFRSF10A (AUC=0.891), ST6GAL1 (AUC=0.855) and CCL18 (AUC=0.854) discriminate RA patients and HDs with high specificity and sensitivity, suggesting the potential role of these proteins to diagnose RA patients. Of note, the combination of all these six proteins could be used as candidate diagnostic biomarkers tool for RA with AUC of 0.970 (**Figure 2A**). In contrast, we compared the levels of autoantibodies and CRP with the combined proteome to assess these new molecular concepts against the standard criteria for the diagnosis of RA. Hence, ROC analysis demonstrated that elevated levels of RF (AUC=0.915) and ACPAs (AUC=0.845) can effectively discriminate RA patients from HDs. In contrast, levels of CRP (AUC=0.572) did not reach statistical significance (**Figure 2B**). Notably, the combined serum proteome profile of CTSL-1, SORT1, SAA4, TNFRSF10A, ST6GAL1, and CCL18 exhibited better ROC curve for distinguishing patients from HDs compared to the combination of ACPAS, RF and CRP (**Figures 2B, C**).

### 3.2 Effects of methotrexate and tofacitinib on the cardiometabolic and CVD-proteome

We conducted a longitudinal study involving 50 RA patients treated with methotrexate or tofacitinib for six months to analyze the impact of these therapies on the proteome related to cardiometabolism and CVD. After six months of treatment, disease activity and CRP levels decreased with both therapies (**Table 2**). The group treated with methotrexate (early RA) had an 80% ratio of responders according to EULAR response criteria, while the group treated with tofacitinib (established RA) had a 72% responder ratio (**Table 2**). No CVD events were observed during the six-month treatment period with either methotrexate or tofacitinib.

**Table 2:** Longitudinal study of early and established Rheumatoid Arthritis patients treated with methotrexate or tofacitinib: clinical details.

Early RA – Methotrexate treatment		
**Disease duration (years)**	0	
**Time**	Basal	6 months
**Size population (n)**	25	
**Female/male (%)**	75/25	
**Age (years)**	61.24 ± 12.65	
**DAS-28**	5.04 ± 1.20	2.94 ± 1.16***
**CRP (mg/ml)**	1.48 ± 1.90	0.78 ± 0.67*
**Responders (%)**	80	
Long established RA – Tofacitinib treatment		
**Disease duration (years)**	37.48 ± 11.79	
**Time**	Basal	6 months
**Size population (n)**	25	
**Female/male (%)**	70/30	
**Age (years)**	63.75 ± 10.19	
**DAS-28**	4.84 ± 0.70	3.33 ± 1.12***
**CRP (mg/ml)**	8.48 ± 7.53	3.51 ± 6.15**
**Responders (%)**	72	

Data are represented by mean ± SD. HDs, Healthy donors; RA, Rheumatoid arthritis, DAS, disease activity score; CRP, c-reactive protein; *Significant differences vs basal time, p value < 0.05; **Significant differences vs basal time, p value < 0.01; ***Significant differences vs basal time, p value < 0.0001.

**Figure 3:**
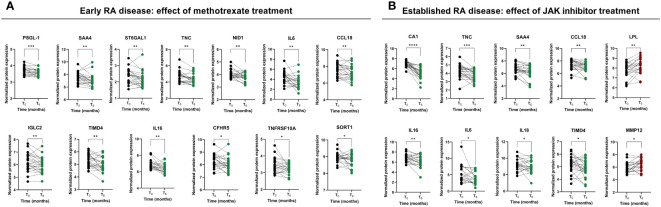
Effect of methotrexate and JAK inhibitor treatments on the serum proteome profile in early and established RA patients, respectively. **(A)** CVD and cardiometabolic-related proteins modulated by the treatment with methotrexate after six months in early RA. **(B)** CVD and cardiometabolic-related proteins modulated by the treatment with methotrexate after six months in established RA. RA, rheumatoid arthritis; CVD, cardiovascular disease; ROC, receiver operating characteristic. Annotated names of abbreviated proteins (**Supplementary Table 1**). Graphs of symbols and lines represent levels of analyzed proteins before and after the treatments. Statistical significance levels were designated as follows: (****) <0.0001 p-value; (***) <0.001 p-value; (**) <0.01 p-value; (*) <0.05 p-value.

**Figure 4:**
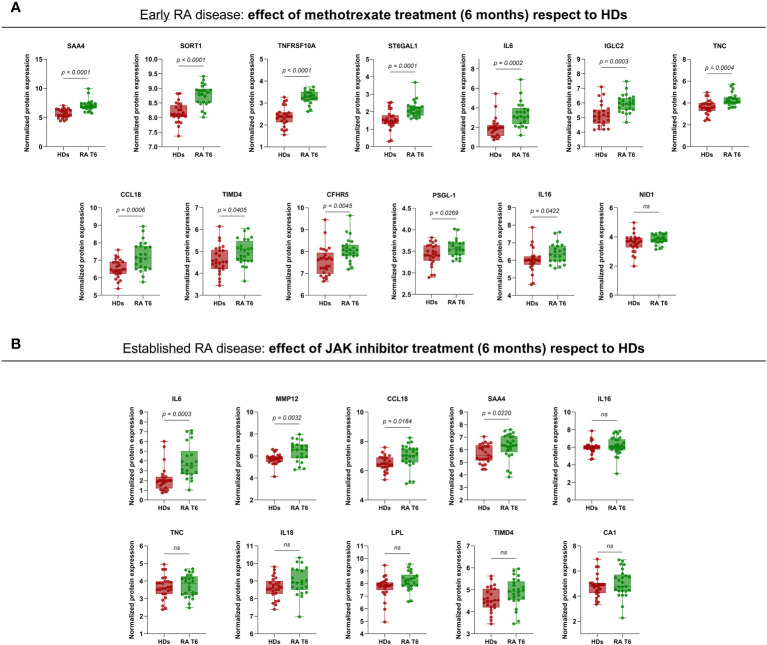
Comparison of serum protein levels at 6 months with levels in healthy individuals. **(A)** Levels of proteins influenced by methotrexate in early RA patients compared to serum levels in healthy individuals. **(B)** Levels of proteins influenced by tofacitinib in established RA patients compared to serum levels in healthy individuals. RA, rheumatoid arthritis; RA T6, rheumatoid arthritis time 6 months; HDs, healthy donors; JAK, janus kinase. Annotated names of abbreviated proteins (**Supplementary Table 1**). Box and whiskers plots represent median and minimum and maximum values of analyzed proteins. The adjusted *p*-values are presented using the Benjamini-Hochberg procedure.

We then examined the impact of treatments on cardiometabolic and CVD-related proteins that were significantly elevated in the serum of RA patients (described in section 3.1). Methotrexate treatment resulted in a significant reduction of the levels of 13 proteins, including SAA4, ST6GAL1, TNC, NID1, SORT1, TNFRSF10A, IL6, CCL18, IGLC2, TIMD4, CFHR5, IL16 and PSGL-1 (**Figure 3A**). On the other hand, tofacitinib significantly reduced the levels of 8 proteins including CA1, TNC, SAA4, CCL18, TIMD4, IL16, IL6 and IL18, and increased levels of 2 proteins such as LPL and MMP12 (**Figure 3B**). Following this, we aimed to gauge the physiological significance of the alterations in protein levels resulting from distinct treatments (methotrexate and tofacitinib). We conducted a comparison of protein levels at the 6-month mark in RA patients to ascertain whether they significantly differed from or mirrored the protein levels in healthy individuals. Among the 13 proteins that were reduced by methotrexate, levels of TIMD4, CFHR5, PSGL-1, IL16 and NID1 were restored at levels seen in HDs (**Figure 4A**). When comparing the levels of proteins influenced by tofacitinib to those in healthy individuals, we detected significant differences in two proteins. In contrast, eight proteins reached levels resembling those observed in healthy individuals (**Figure 4B**), underscoring the potential of tofacitinib to revert the observed alterations in established RA patients.

**Figure 5:**
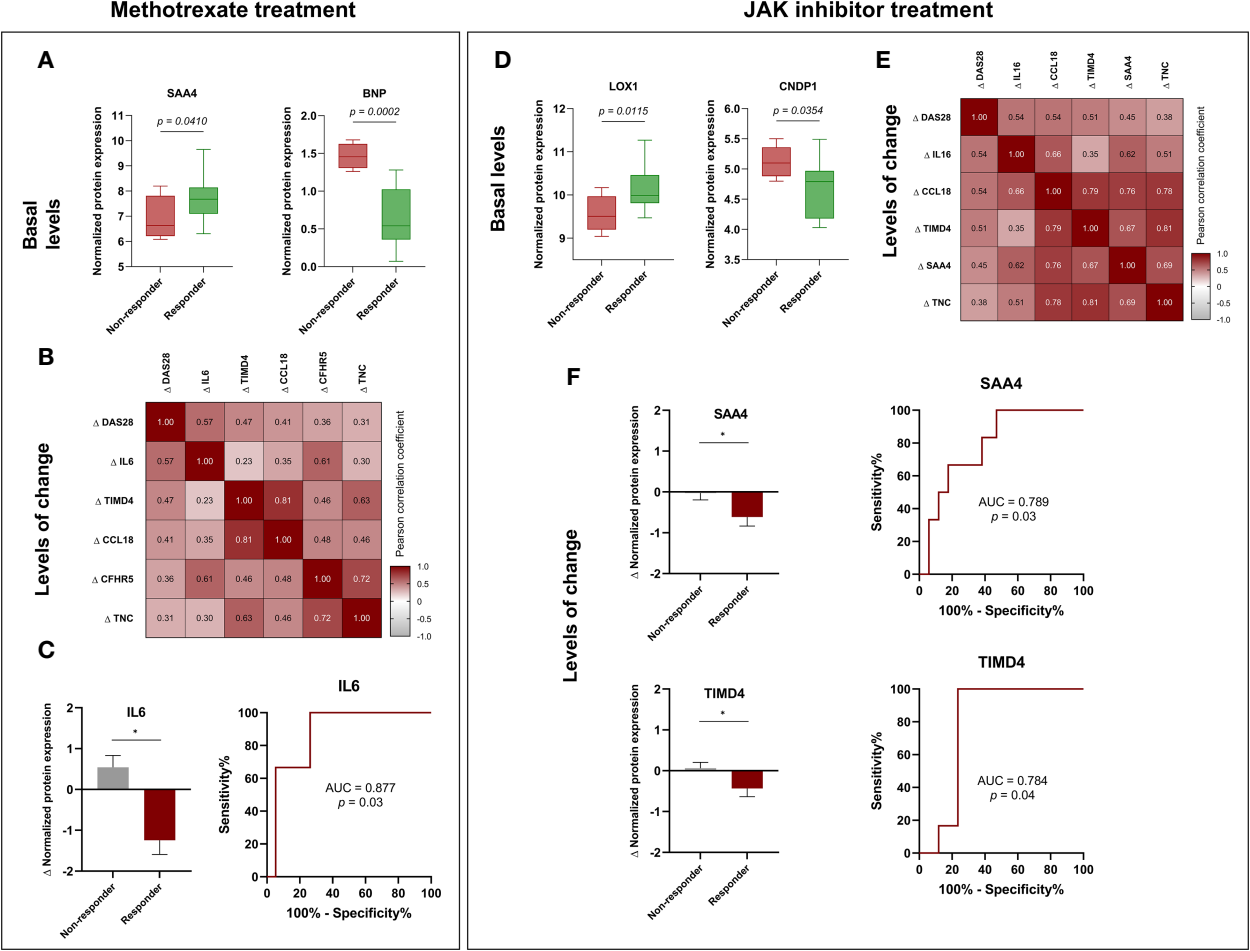
Potential biomarkers for predicting response to methotrexate in early RA and JAK inhibitor treatment in established RA. **(A)** Baseline levels of SAA4 and BNP in methotrexate-treated patients: a comparison between responders and non-responders. **(B)** Correlation heatmap of changes in DAS28 and proteins after six months of treatment with methotrexate. **(C)** Comparison of changes (Δ) in IL6 levels in responders and non-responders RA patients and ROC curve of changes in IL6 levels to discriminate responders or non-responders RA patients after six months of treatment with methotrexate. **(D)** Baseline levels of LOX1 and CNDP1 in JAK inhibitor-treated patients: a comparison between responders and non-responders. **(E)** Correlation heatmap of changes (Δ) in DAS28 and proteins after six months of treatment with JAK inhibitor. **(F)** Comparison of changes (Δ) in SAA4 and TIMD4 levels in responders and non-responders RA patients after the treatment with JAK inhibitor and ROC curve analyses. RA, rheumatoid arthritis; ROC, receiver operating characteristic. Annotated names of abbreviated proteins (**Supplementary Table 1**). Box and whiskers plots represent median and minimum and maximum values of basal proteins. Correlation heatmap represent significant correlations between disease activity and proteins. Numbers in correlation heatmaps include Pearson correlation coefficient (r). Bar graphs represent mean with standard deviation (error bars). *Significant differences: *p*<0.05. The adjusted *p*-values for the box plots did not demonstrate statistical significance, except in the case of the BNP protein, where the adjusted *p*-value was 0.037.

Moreover, we investigated the potential association between baseline protein levels or alterations during methotrexate treatment and the response to therapy. Intriguingly, elevated levels of SAA4 and reduced levels of BNP at the treatment’s onset were associated with a positive treatment response (**Figure 5A**). Conversely, changes in the levels of IL6, TIMD4, CCL18, CFHR5, and TNC following methotrexate treatment were identified as linked to alterations in DAS28, indicating their association with disease activity (**Figure 5B**). Notably, changes in IL6 levels were specifically linked to the response to methotrexate, with RA responder patients displaying significantly decreased IL6 levels post-treatment compared to non-responder patients. The changes in IL6 levels demonstrated the potential to discriminate between responder and non-responder patients, achieving an AUC of 0.877 (**Figure 5C**). Upon transitioning to tofacitinib treatment, discernible differences in baseline levels of LOX1 and CNDP1 were noted between non-responder and responder RA patients (**Figure 5D**). Subsequent analyses revealed that changes in the levels of IL16, CCL18, TIMD4, SAA4, and TNC significantly correlated with alterations in DAS28 (**Figure 5E**), implying their association with disease activity. Thus, decreased levels of SAA4 or TIMD4 were found in RA responder patients compared to the non-responder group (**Figure 5F**). Annotated names of abbreviated proteins are stated in **Supplementary Table 1**.

## 4 Discussion

**Figure 6:**
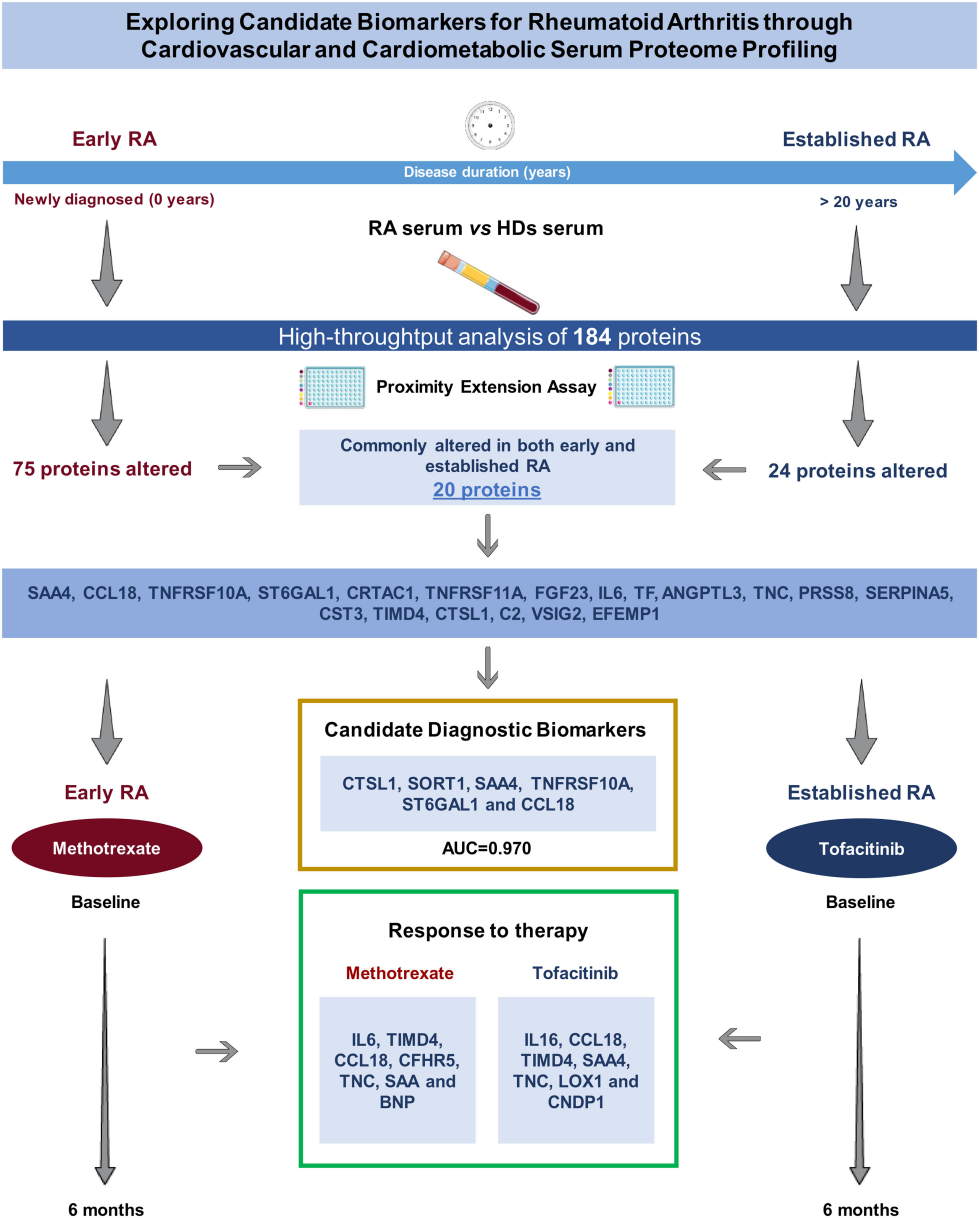
Study summary: A high-throughput analysis of 184 proteins across diverse stages of RA (early and established disease) uncovered a consistently altered pattern, indicating potential diagnostic biomarkers, including CTSL1, SORT1, SAA4, TNFRSF10A, ST6GAL1, and CCL18. Methotrexate was administered to early RA patients, while tofacitinib was employed for established RA cases. Post a 6-month treatment period, the levels of proteins exhibiting alterations in both early and established RA were modulated, pinpointing susceptible proteins that may serve as candidate biomarkers indicative of the response to therapy. Annotated names of abbreviated proteins (**Supplementary Table 1**). RA, rheumatoid arthritis; HDs, healthy donors; AUC, area under the curve.

This study describes molecular alterations that might be associated to cardiometabolic and cardiovascular disease in the serum of RA patients, using a high-throughput proteomic technology to analyze the serum levels of 184 proteins (**Figure 6**). Our work identifies novel potential candidate biomarkers of RA diagnosis and therapeutic targets including CTSL-1, SORT1, SAA-4, TNFRSF10A, ST6GAL1 and CCL18, in two different cohort of RA patients. Additionally, it is showed how methotrexate or JAK-inhibitor can modulate these protein alterations and detects biomarkers of response to each therapy. With the use of the PEA technology, we were able to directly analyze a substantial number of proteins that has been described to be involved in cardiovascular and cardiometabolic diseases in the serum of patients with RA. We found 75 proteins significantly altered in the serum of RA patients with early disease, 20 of them were also elevated in RA patients with established disease. These findings may suggest an association of RA with a modification of the molecular profile that might be related to CVD, which appears elevated in patients recently diagnosed and persisted over an extended period of evolution. In a previous study conducted by Ferreira and colleagues, a panel of 92 proteins related to cardiovascular disease (Olink proteomics) was assessed in RA patients who had been suffering from the disease for a long period of time and 6.8% of them had heart failure (HF) diagnosis. They identified some biomarkers that were associated with HF (10). Interestingly, some of those proteins increased in patients with HF were also increased in our cohort of RA patients, especially in the cohort of established disease, such as PGF, TNFRSF10A, SPON-2, TF and PRSS8. In our work, we discovered six proteins that exhibit promising potential as biomarkers for the differentiation of individuals with RA from HDs. Specifically, proteins exhibiting alterations in two distinct cohorts of RA patients with both early and established disease, including CTSL-1, SORT1, SAA4, TNFRSF10A, ST6GAL1 and CCL-18. It is noteworthy that the combined use of these six proteins resulted in an improved area under the curve (AUC) of 0.97. In addition, this combined proteome signature exhibited better AUC compared to the performance of stablished biomarkers of RA, such as ACPAs, RF and CRP alone and combined. The data presented herein corroborates recent findings indicating that SAA-4 may serve as a promising serum biomarker for the diagnosis of RA, including cases of seronegative presentation (11–13). ST6GAL1 is a pivotal sialyltransferase enzyme responsible for catalyzing the addition of α2,6-linked sialic acids to glycans’ termini. Glycosylation with sialic acid is a notable modification for IgGs. This glycosylation process has been acknowledged for its immunoregulatory impact on various immune cells, including stem cells, B cells, T cells, and macrophages (14). While ST6GAL1 is responsible for adding sialic acid to glycoproteins, the overall sialylation of IgG has been shown reduced in RA patients (15). The elevated levels of ST6GAL1 that we found in the serum of RA patients might be associated with increased inflammatory responses. The reduction in sialylated IgG could be due to several factors independent on the levels of ST6GAL1, such as increased turnover or degradation of sialylated IgG, altered glycosylation patterns driven by other enzymes, or changes in the microenvironment that affect the glycosylation process. In addition, we measured levels of protein, not enzyme activation. Thus, the elevation of ST6GAL1 levels in the serum of RA patients and the reduction in sialylated IgG may be part of the complex molecular and cellular changes associated with RA. Further research is needed to elucidate the precise mechanisms and functional consequences of these alterations.

The role of CCL18 in the pathogenesis of RA was investigated some time ago, with studies demonstrating a substantial elevation in the serum levels of this protein among RA patients in comparison with HDs. Moreover, these studies also revealed a positive correlation between the levels of CCL18 and the activity of the disease (16).

TNFRSF10A, also known as TRAIL-R1, presents a complex role in the context of RA synovial fibroblasts, exhibiting dual effects. While it is acknowledged for inducing apoptosis through caspase activation upon TRAIL ligand binding (17), its interaction with other receptors, including TNFRSF10A/TRAIL-R1, can activate NFκB, a transcription factor regulating cell proliferation (18). Morel et al. reported TRAIL’s ability to stimulate RA fibroblast proliferation *in vitro* via MAP kinase and PI3 kinase/Akt activation (19). Paradoxically, in RA patients, both serum TRAIL and IL-8 concentrations were elevated, while TRAIL receptor expression, including TNFRSF10A/TRAIL-R1, was reduced in monocytes. This led to decreased TRAIL-induced monocyte apoptosis in RA due to increased TRAIL-induced IL-8 secretion, activating antiapoptotic pathways (20). These findings underscore the potential pathogenic role of TNFRSF10A in RA. Moreover, SORT1, a pivotal regulator of lipid metabolism (21), has been previously noted to be elevated in the serum of RA patients compared to healthy donors. It plays a critical role in immune cell signaling, contributing to the pathogenesis of RA (22). On the other hand, CTSL1, an endosomal proteolytic enzyme involved in extracellular matrix degradation, angiogenesis, and antigen presentation, is elevated in the circulation of RA patients, particularly associated with autoantibodies (23, 24). Our study affirms that TNFRSF10A, SORT1 and CTSL1 are significantly elevated not only in early RA but also in established disease, suggesting their potential as candidate biomarkers for the disease.

The effects of methotrexate therapy on cardiometabolic and cardiovascular alterations in RA patients have yielded inconsistent results in prior studies (25, 26). In our study of patients with early RA disease, we observed that the administration of methotrexate over a six-month period resulted in a significant reduction of 13 molecules that have been related to CVD. Although this reduction did not reach the levels of these proteins in healthy subjects. These results suggest that methotrexate might have beneficial effects on the molecular profile related to CVD. Currently, one of the areas that have garnered considerable attention is the identification of biomarkers indicative of the response to therapy among patients diagnosed with RA. This pursuit is primarily aimed at early disease progression, improvement of patient outcome, reducing healthcare costs and promoting a better understanding of the disease. Numerous studies have been conducted to identify clinical or molecular factors or a combination of both, that could predict the effectiveness of methotrexate therapy. The most recent studies have explored the integration of genomics and clinical data through machine learning to predict the response to methotrexate. As such, machine learning methods, which incorporate demographic data, smoking habit, rheumatoid factor, DAS28 and 160 SNPs predicted methotrexate response at 3 months with an AUC of 0.84 (27). With regard to serum biomarkers, prior studies have indicated that four specific proteins may be predictive of methotrexate response in a cohort of RA patients. Notably, high levels of these biomarkers, namely CRP, leptin, TNF-RI and VCAM-1, were found to be associated with low disease activity at 3 months (28). Our research contributes to the biomarker field by identifying serum levels of IL6, SAA4 or BNP as potential predictors of response to methotrexate. It should be emphasized that further research is necessary to confirm these findings and to establish the predictive value of these biomarkers in this regard.

On the other hand, the impact of tofacitinib on CVD risk among RA patients has been a subject of inquiry (29–31). Nonetheless, a considerable cohort study indicated a minimal occurrence of CV events associated with tofacitinib therapy in RA patients (22). A recent study using PEA (Olink proteomics) on a small cohort of RA patients, presented evidence of the modulatory effects of tofacitinib on various inflammatory-related circulating proteins (32). Our study reveals that tofacitinib modulates the levels of 10 proteins potentially associated with CVD in RA patients with established disease. Notably, in six of these proteins, the reduction reached levels comparable to those observed in healthy donors. In this sense, CA1 belongs to the carbonic anhydrase (CA) family, catalyzing reversible hydration and dehydration reactions of CO2/H2CO3 and also can promote the formation of CaCO3. In our study, RA patients with established disease exhibited higher levels than those in the early stages of the disease. Notably, treatment with tofacitinib significantly reduced these elevated levels reaching the levels observed in HDs. Our findings align with a study conducted by Zheng and colleagues, demonstrating that overexpression of CA1 accelerated joint inflammation and tissue destruction in a collagen-induced arthritis (CIA) mice model (33). This supports our observation that RA patients with established disease may exhibit higher CA1 levels than those in the early stages of RA. Furthermore, recent research has positioned CA1 as a potential target for managing pain symptoms associated with RA and related inflammatory diseases (34, 35). Our study contributes to the growing body of evidence linking CA1 to RA pathogenesis and treatment response, emphasizing its potential as a therapeutic target in the management of RA. These results could also suggest CA1 as a molecular target for tofacitinib, with the potential to beneficially reduce pain.

Insufficient evidence exists concerning the predictors of clinical response in RA patients undergoing tofacitinib treatment. Recently, a study of 25 RA patients indicated that baseline power Doppler and multi-biomarker disease activity (MBDA) score can forecast the response to tofacitinib (36). Furthermore, early alterations in magnetic resonance may serve as a predictor of the response to therapy, either to methotrexate or tofacitinib (37). Nonetheless, there is an absence of data on molecular predictors of the response to tofacitinib treatment. Our preliminary study reveals novel findings regarding the serum levels of LOX1, CNDP1, TIMD4 and SAA4 as biomarkers of the response to this therapy.

### 4.1 Limitations of the study

A key limitation of this study was its small sample size and the lack of randomization in the longitudinal cohort. The non-randomized recruitment from routine clinical practice may have led to uneven distribution among treatment groups, potentially affecting therapy response. Consequently, our results only allow us to make predictive statements about the link between serum proteins and methotrexate or tofacitinib treatment responses. Therefore, larger validation cohorts using complementary techniques are essential to validate these findings.

## 5 Conclusions

This study aimed to assess the alterations in the serum proteome related to CVD in two cohorts of active rheumatoid arthritis (RA) patients: those who were newly diagnosed (naïve-treated) and those with well-established disease. Our findings revealed that RA is characterized by a distinct and modified CVD proteome, which was observed both at early stages of the disease and throughout its extended progression. This indicates that the alteration in the proteome is a persistent feature of RA. Moreover, we identified a six-biomarker serum panel consisting of CTSL-1, SORT1, SAA4, TNFRSF10A, ST6GAL1, and CCL-18 proteins that effectively distinguished the two cohorts of RA patients from healthy donors. These biomarkers hold potential as valuable candidate diagnostic indicators for RA. Additionally, we observed distinct impacts of methotrexate and tofacitinib on the levels of these proteins, with each treatment modifying a diverse set of proteins. Our results demonstrated that tofacitinib response was associated with baseline levels of LOX1, CNDP1 and changes in SAA4 and TIMD4 proteins. Conversely, methotrexate response showed an association with changes in IL-6 protein levels and the basal levels of SAA4 and BNP. In summary, our study provides insights into the altered proteomic profile associated with CVD in RA patients and identifies a promising six-biomarker panel for RA diagnosis. Furthermore, we elucidated the specific modulation of these proteins by methotrexate and tofacitinib, highlighting potential avenues for personalized treatment strategies in RA management.

## Data availability statement

The original contributions presented in the study are included in the article/**Supplementary Materials**, further inquiries can be directed to the corresponding author/s.

## Ethics statement

The studies involving humans were approved by ethics committee of the Reina Sofia Hospital, the University Clinical Hospital of Santiago de Compostela, and La Paz University Hospital. The studies were conducted in accordance with the local legislation and institutional requirements. The participants provided their written informed consent to participate in this study.

## Author contributions

LC-L: Data curation, Formal Analysis, Investigation, Methodology, Writing – original draft. AE-C: Funding acquisition, Project administration, Resources, Supervision, Writing – review & editing. YH: Data curation, Formal Analysis, Software, Writing – original draft. CP-S: Investigation, Methodology, Writing – review & editing. MR-P: Investigation, Methodology, Writing – original draft. JMM-M: Investigation, Methodology, Writing – original draft. EP-P: Methodology, Writing – original draft. AG: Methodology, Writing – review & editing. CP-R: Methodology, Writing – review & editing. AM-F: Methodology, Writing – review & editing. AB: Methodology, Writing – review & editing. CL-M: Data curation, Formal Analysis, Writing – review & editing. LL-P: Methodology, Writing – review & editing. MR-G: Methodology, Writing – review & editing. RO-C: Methodology, Writing – review & editing. JC-G: Methodology, Writing – review & editing. CL-P: Writing – review & editing. EC-E: Data curation, Formal Analysis, Writing – review & editing. IA-R: Writing – original draft. NB: Funding acquisition, Investigation, Project administration, Resources, Supervision, Writing – original draft, Writing – review & editing.

## Supplementary material

Supplementary Figure 1 Comparison of the commonly altered serum proteome in early and established RA compared to HDs. Box and whiskers plots represent median and minimum and maximum values of analyzed proteins. One-way ANOVA and Bonferroni’s *post hoc* multiple comparisons tests were conducted for statistical analysis. (****) <0.0001 adj-p-value; (***) <0.001 adj-p-value; (**) <0.01 adj-p-value; (*) <0.05 adj-p-value. RA: rheumatoid arthritis; HDs: healthy donors. Annotated names of abbreviated proteins (**Supplementary Table 1**).
