# Supplementary material for: Exploring candidate biomarkers for rheumatoid arthritis through cardiovascular and cardiometabolic serum proteome profiling
Source: Front Immunol. 2024 Feb 14;15:1333995. doi: 10.3389/fimmu.2024.1333995 (PMC10900234; doi:10.3389/fimmu.2024.1333995)
Supplement: Supplementary file 2 [file Table_1.docx]

**Supplementary table I**. Annotated names of abbreviated proteins.

| **Abbreviation** | **Annotated name** |
| --- | --- |
| ACE2 | Angiotensin Converting Enzyme 2 |
| AGRP | [Agouti-related protein](https://www.olink.com/products-services/target/protein/?assayID=4649) |
| ANG | Angiogenin |
| ANGPTL3 | [Angiopoietin-related protein 3](https://www.olink.com/products-services/target/protein/?assayID=4488) |
| BNP | Natriuretic Peptide B |
| BOC | BOC Cell Adhesion Associated, Oncogene Regulated |
| C1QTNF1 | C1q And TNF Related 1 |
| C2 | Complement C2 |
| CA1 | [Carbonic anhydrase 1](https://www.olink.com/products-services/explore/protein/?proteinID=OID20409) |
| CCL18 | [C-C motif chemokine 18](https://www.olink.com/products-services/target/protein/?assayID=4446) |
| CCL14 | chemokine (C-C motif) ligand 14 |
| CCL5 | C-C Motif Chemokine Ligand 5 |
| CFHR5 | [Complement factor H-related protein 5](https://www.olink.com/products-services/target/protein/?assayID=4490) |
| CHL1 | Cell Adhesion Molecule L1 Like |
| CNDP1 | Carnosine Dipeptidase 1 |
| COMP | Cartilage Oligomeric Matrix Protein |
| CRTAC1 | Cartilage Acidic Protein 1 |
| CST3 | [Cystatin-C](https://www.olink.com/products-services/target/protein/?assayID=4469) |
| CTSL1 | [Cathepsin L1](https://www.olink.com/products-services/target/protein/?assayID=4695) |
| DECR1 | 2,4-Dienoyl-CoA Reductase 1 |
| EFEMP1 | [EGF-containing fibulin-like extracellular matrix protein 1](https://www.olink.com/products-services/target/protein/?assayID=4443) |
| F7 | Coagulation Factor VII |
| FCGR2A | Fc Gamma Receptor IIa |
| FCGR3B | Fc Gamma Receptor IIIb |
| FGF-23 | Fibroblast Growth Factor 23 |
| GAL-9 | [Galectin-9](https://www.olink.com/products-services/target/protein/?assayID=4620) |
| GAS6 | Growth Arrest Specific 6 |
| GIF | Gastric Intrinsic Factor |
| GNLY | Granulysin |
| GT | Gastrotropin |
| hOSCAR | Osteoclast Associated Ig-Like Receptor |
| ICAM1 | [Intercellular adhesion molecule 1](https://www.olink.com/products-services/target/protein/?assayID=4436) |
| ICAM3 | [Intercellular adhesion molecule 3](https://www.olink.com/products-services/target/protein/?assayID=4450) |
| IGLC2 | [Immunoglobulin lambda constant 2](https://www.olink.com/products-services/target/protein/?assayID=4470) |
| IL16 | [Pro-interleukin-16](https://www.olink.com/products-services/target/protein/?assayID=4652) |
| IL-17D | Interleukin 17D |
| IL18 | Interleukin 18 |
| IL-1RA | Interleukin 1 Receptor Antagonist |
| IL-27 | Interleukin 27 |
| IL-4RA | Interleukin-4 Receptor Alpha Chain |
| IL6 | [Interleukin-6](https://www.olink.com/products-services/explore/protein/?proteinID=OID20101) |
| LILRB1 | Leukocyte Immunoglobulin Like Receptor B1 |
| LILRB2 | Leukocyte Immunoglobulin Like Receptor B2 |
| LOX1 | [Oxidized low-density lipoprotein receptor 1](https://www.olink.com/products-services/target/protein/?assayID=4625) |
| LPL | [Lipoprotein lipase](https://www.olink.com/products-services/explore/protein/?proteinID=OID20188) |
| MEGF9 | Multiple EGF Like Domains 9 |
| MERTK | [Tyrosine-protein kinase Mer](https://www.olink.com/products-services/target/protein/?assayID=4594) |
| MET | [Hepatocyte growth factor receptor](https://www.olink.com/products-services/explore/protein/?proteinID=OID20269) |
| MFAP5 | Microfibril Associated Protein 5 |
| MMP12 | [Macrophage metalloelastase](https://www.olink.com/products-services/target/protein/?assayID=4663) |
| MMP7 | [Matrilysin](https://www.olink.com/products-services/target/protein/?assayID=4648) |
| NID1 | [Nidogen-1](https://www.olink.com/products-services/target/protein/?assayID=4409) |
| NRP1 | Neuropilin 1 |
| OSMR | Oncostatin M Receptor |
| PAM | Peptidylglycine Alpha-Amidating Monooxygenase |
| PAR-1 | Proteinase-Activated Receptor 1 |
| PCOLCE | Procollagen C-Endopeptidase Enhancer |
| PGF | [Placenta growth factor](https://www.olink.com/products-services/target/protein/?assayID=5579) |
| PLTP | Phospholipid Transfer Protein |
| PLXNB2 | [Plexin-B2](https://www.olink.com/products-services/target/protein/?assayID=4429) |
| PROC | Protein C, Inactivator Of Coagulation Factors Va And VIIIa |
| PRSS8 | [Prostasin](https://www.olink.com/products-services/target/protein/?assayID=4645) |
| PSGL-1 | [P-selectin glycoprotein ligand 1](https://www.olink.com/products-services/target/protein/?assayID=4688) |
| QPTC | Glutaminyl-Peptide Cyclotransferase |
| REN | Renin |
| SAA4 | [Serum amyloid A-4 protein](https://www.olink.com/products-services/explore/protein/?proteinID=OID30726) |
| SERPINA5 | [Plasma serine protease inhibitor](https://www.olink.com/products-services/target/protein/?assayID=4471) |
| SOD2 | Superoxide Dismutase 2 |
| SORT1 | [Sortilin](https://www.olink.com/products-services/target/protein/?assayID=4685) |
| ST6GAL1 | [Beta-galactoside alpha-2,6-sialyltransferase 1](https://www.olink.com/products-services/target/protein/?assayID=4411) |
| TCN2 | Transcobalamin 2 |
| TF | [Tissue](https://www.olink.com/products-services/explore/protein/?proteinID=OID30783) Factor |
| TIMD4 | T Cell Immunoglobulin And Mucin Domain Containing 4 |
| TIMP1 | TIMP Metallopeptidase Inhibitor 1 |
| TNC | [Tenascin](https://www.olink.com/products-services/target/protein/?assayID=4425) |
| TNFRSF10A | [Tumor necrosis factor receptor superfamily member 10A](https://www.olink.com/products-services/target/protein/?assayID=4644) |
| TNFRSF11A | TNF Receptor Superfamily Member 11a |
| TRAIL-R2 | [Tumor necrosis factor receptor superfamily member 10B](https://www.olink.com/products-services/target/protein/?assayID=4641) |
| VCAM1 | [Vascular cell adhesion protein 1](https://www.olink.com/products-services/target/protein/?assayID=4416) |
| VSIG2 | V-Set And Immunoglobulin Domain Containing 2 |
| XCL1 | X-C Motif Chemokine Ligand 1 |
